# Epitaxial growth of Cu(001) thin films onto Si(001) using a single-step HiPIMS process

**DOI:** 10.1038/s41598-017-01755-8

**Published:** 2017-05-10

**Authors:** Felipe Cemin, Daniel Lundin, Clarisse Furgeaud, Anny Michel, Guillaume Amiard, Tiberiu Minea, Gregory Abadias

**Affiliations:** 1 Laboratoire de Physique des Gaz et des Plasmas (LPGP), UMR 8578 CNRS, Université Paris-Sud, Université Paris-Saclay, 91405 Orsay, France; 2 Institut Pprime, Département Physique et Mécanique des Matériaux, UPR 3346 CNRS, Université de Poitiers, 86962 Chasseneuil-Futuroscope, France

## Abstract

We report on a new route to grow epitaxial copper (Cu) ultra-thin films (up to 150 nm thick) at ambient temperature on Si(001) wafers covered with native oxide without any prior chemical etching or plasma cleaning of the substrate. It consists of a single-step deposition process using high power impulse magnetron sputtering (HiPIMS) and substrate biasing. For a direct current (DC) substrate bias voltage of −130 V, Cu/Si heteroepitaxial growth is achieved by HiPIMS following the Cu(001) [100]//Si(001) [110] orientation, while under the same average deposition conditions, but using conventional DC magnetron sputtering, polycrystalline Cu films with [111] preferred orientation are deposited. In addition, the intrinsic stress has been measured *in situ* during growth by real-time monitoring of the wafer curvature. For this particular HiPIMS case, the stress is slightly compressive (−0.1 GPa), but almost fully relaxes after growth is terminated. As a result of epitaxy, the Cu surface morphology exhibits a regular pattern consisting of square-shaped mounds with a lateral size of typically 150 nm. For all samples, X-ray diffraction pole figures and scanning/transmission electron microscopy reveal the formation of extensive twinning of the Cu {111} planes.

## Introduction

The crystallographic orientation of thin films is of great importance in the performance of advanced electronic, optoelectronic, magnetic and superconducting heterostructures and devices, especially when the thickness of the films is reduced to the nanometer scale^1^. For instance, the magnetization phenomena governing magneto-optical recording and spin polarized devices is anisotropic, *i*.*e*., it preferentially occurs in a certain crystallographic orientation of the deposited magnetic layer^2, 3^. For such devices, ultrathin epitaxial metallic layers are usually deposited on semiconductor substrates to enable a particular growth direction for subsequent deposition of magnetic thin films (acting as a *seed layer*)^3–7^ or to reduce the dislocation density of lattice mismatched heterostructures (acting as a *buffer layer*)^1, 7, 8^. Copper (Cu) films epitaxially grown on silicon (Si) substrates have been extensively studied^9–18^ and used for these purposes^3–7^ due to the low electrical resistivity of Cu as well as its high electromigration resistance^19^. However, as previously reported, the heteroepitaxial growth of Cu on Si can only be achieved by surface atomic cleaning processes to eliminate native oxides and contaminants of the substrate prior to deposition. Standard pre-treatment methods include heating of the substrate at relatively high temperatures (800 °C)^10, 12, 13, 20^ and surface chemical etching with hydrofluoric acid (HF), which creates a passivated surface with hydrogen termination on the Si dangling bonds^9–18^. The latter case is the most widespread method used in the past years, even though it is a toxic and time-consuming two-step solution.

In plasma-based deposition technology, ion bombardment in argon or hydrogen glow discharges is commonly used as a first step pre-treatment process to provide crystalline surfaces free of contaminants and native oxides for metallic and semiconductor substrates^21, 22^. More recently, highly ionized metal fluxes were alternatively employed as a pre-treatment using high power impulse magnetron sputtering (HiPIMS)^23, 24^ and cathodic arc^25^ processes combined with a high negative direct current (DC) bias voltage applied to the substrate. This new approach is capable of producing interfaces with a well-defined chemistry, which improves the film/substrate adhesion (compared to the results obtained using conventional Ar glow discharges) and promotes local epitaxial growth of subsequent deposited ceramic layers over large areas^23–25^. Aissa *et al*.^26^ have observed local epitaxial growth of aluminum nitride films on the interface of Si (100) substrates during the early stages of thin film deposition using HiPIMS. Although epitaxial growth throughout the entire thin film has so far not been achieved using the above outlined strategies, these results indicate that the ion bombardment generated by ionized physical vapor deposition methods, such as HiPIMS discharges, can completely, or partially, eliminate the native oxides present on the substrate surface, and ultimately lead to a preferential growth direction at the interface film/substrate.

In this work, we report on a novel single-step, HiPIMS-based deposition process, controlled by DC biasing the substrate, to grow epitaxial Cu thin films up to 150 nm thick on Si (001) oriented wafers covered with native oxide, without any pretreatment process. We discuss the unique HiPIMS process conditions, which were used to go beyond previously published results on epitaxial growth at the substrate/film interface. The HiPIMS Cu films are also compared to reference films deposited at the same experimental conditions by conventional direct current magnetron sputtering (DCMS).

## Results and Discussion

**Figure 1:**
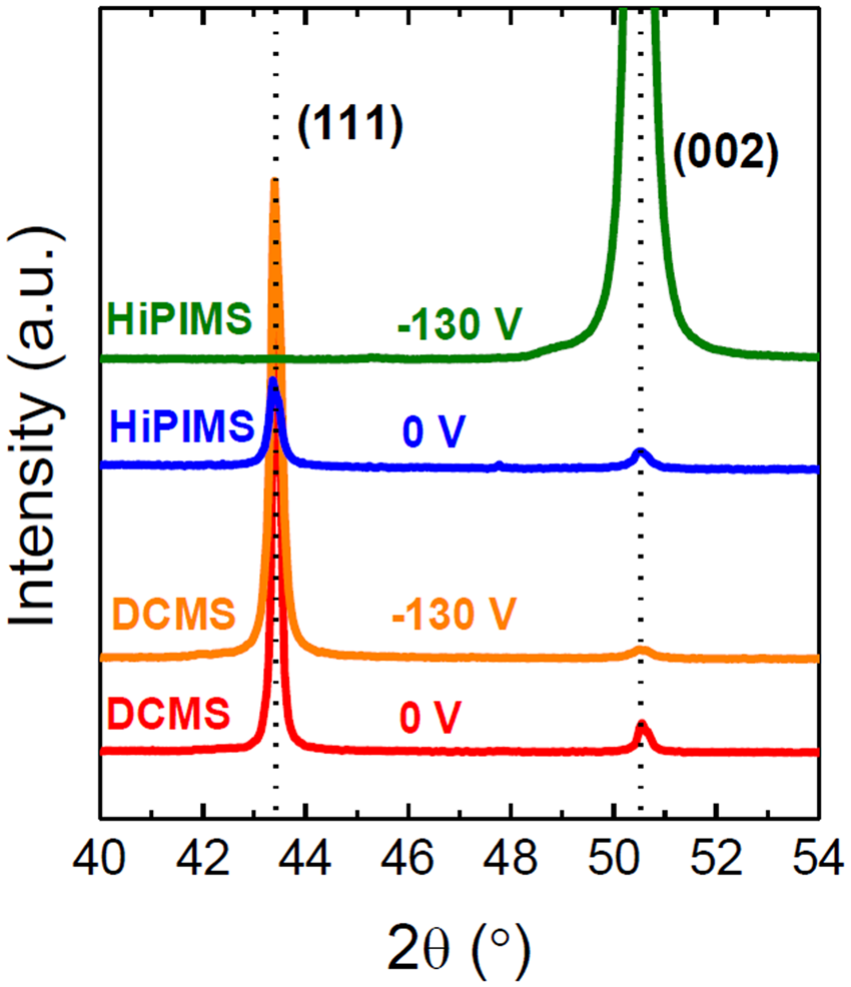
Effect of bias voltage on the film crystal structure. XRD θ–2θ scans for grounded (0 V) and −130 V bias DCMS and HiPIMS Cu films (~150 nm thick).

Figure 1 shows representative θ–2θ X-ray diffraction (XRD) scans of Cu films ~150 nm thick, deposited by DCMS and HiPIMS at a bias voltage of 0 V (grounded substrate) and −130 V (biased substrate), over an angular range covering the two main 111 and 200 Bragg reflections of face centered cubic (fcc) Cu. For DCMS conditions, the (111) preferred orientation is observed, independently of the applied bias voltage. With increasing bias voltage, the full width at half maximum (FWHM) of the 111 XRD line intensity increases from 0.19° to 0.24°, accompanied by a decrease in intensity of the 200 XRD line. For the HiPIMS series, the Cu film deposited at 0 V bias is also characterized by a (111) preferred orientation, as in the DCMS case. However, a noticeable change is seen when the substrate bias voltage reaches −130 V: the XRD pattern exhibits a strong increase of the 200 line intensity at 2θ = 50.3°. At the same time, a significant peak broadening occurs (FWHM of 0.45°), which is attributed to increased microstrain due to energetic ion bombardment conditions, as discussed below.

**Figure 2:**
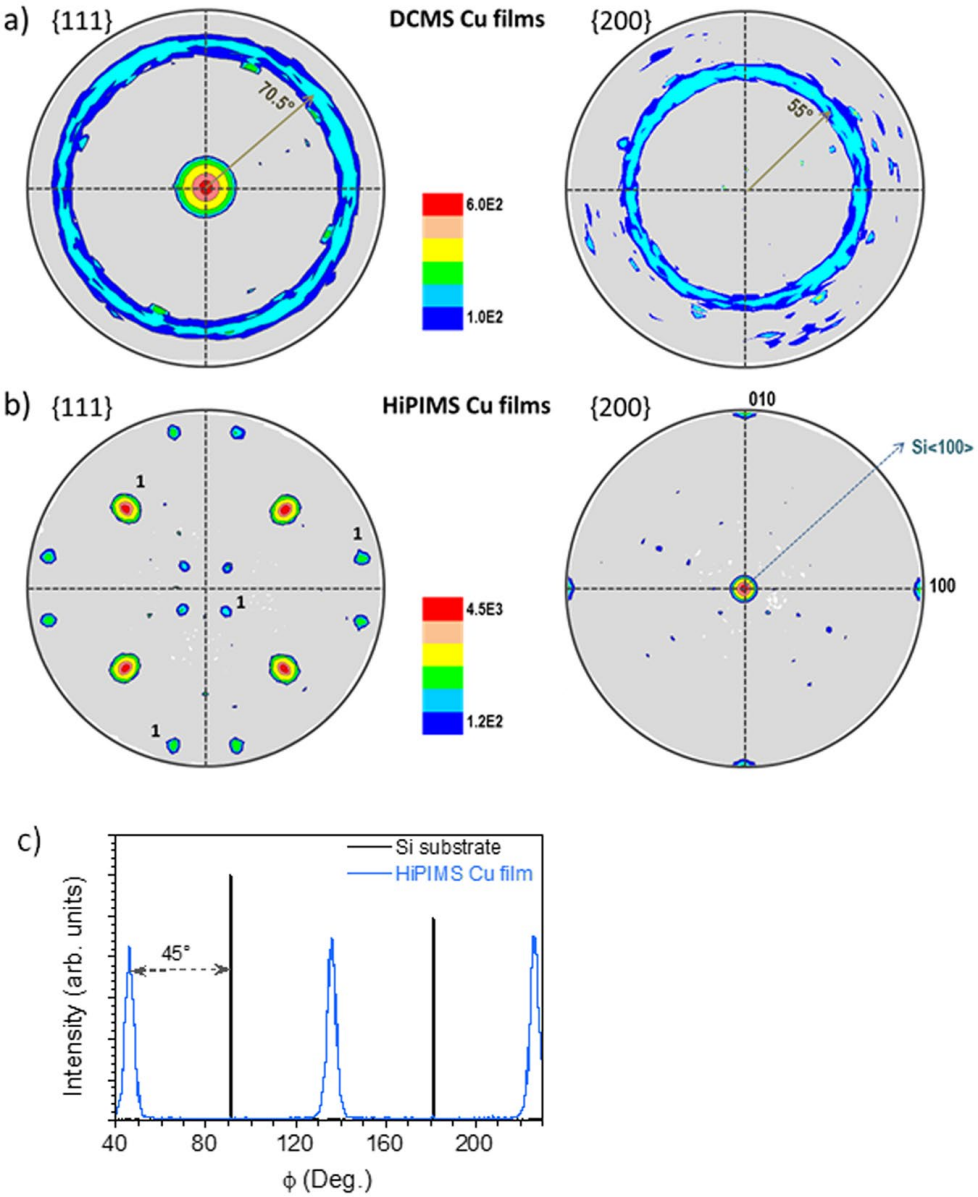
{111} and {200} XRD pole figures of Cu films grown at −130 V bias. (**a**) DCMS film and (**b**) HiPIMS film (both ~150 nm thick). Label 1 denotes poles corresponding to one of the four twin sets associated with ﻿Cu {111} planes. (**c**) Phi-scans of the 111 intensity variation for both HiPIMS Cu film at −130 V and Si substrate, recorded at ψ = 54.74°.

To understand this texture change, XRD pole figures were measured. The {111} and {200} pole figures for the Cu films ~150 nm thick deposited at −130 V bias are displayed in Fig. 2(a) (DCMS film) and Fig. 2(b) (HiPIMS film). For the DCMS film, the 111 intensity maxima are distributed at the center and along a ring located at ψ = 70.5°, while a 200 diffraction ring is found at ψ = 55°, indicating the presence of a polycrystalline film with 111 fiber-texture. This is typically expected for fcc metal films deposited by DCMS on amorphous substrates: crystal nucleation occurs randomly along the azimuthal ϕ direction but as the (111) planes offer the lowest surface energy, 111-oriented islands are energetically favored^27^. However, for the HiPIMS film, both {111} and {200} pole figures display intensity maxima with a four-fold symmetry, in addition to the main pole intensity at the center of the {200} pole figure. These diffraction spots correspond to pole intensity characteristic of an fcc single-crystal oriented along the [001] axis. This indicates that the HiPIMS Cu film deposited at −130 V is not any more fiber-textured, but has grown epitaxially on the Si (001) surface. As indicated in Fig. 2(b), the Cu < 100 > directions are rotated by 45° with respect to the Si < 100 > directions, *i.e.*, the [100] axis of the Cu is parallel to the [110] of the Si. This rotation significantly reduces the lattice mismatch of Cu on Si from 33% to 6%, making epitaxial growth more likely^11^. The epitaxial relationship with the Si substrate is Cu [100] (001) // Si [110] (001), as also illustrated by the ϕ-scans in Fig. 2(c) recorded for the 111 Bragg reflection at ψ = 54.74°. This epitaxial relationship explains the presence of the four 111 maxima at ψ = 54.74° on the {111} pole figure (Fig. 2(b)). However, one can observe additional spots at ψ = 15.8 and 79.0°. These corresponds to twin defects on {111} planes. Three twinning variants, rotated at 120° from each other (see Fig. 2(b)) are formed under the present deposition conditions, as also reported by Chen *et al*.^18^ for epitaxial Cu films on Si substrates. SEM observations (not reported here) confirm the presence of twin defects in the Cu grains. From the diffraction data presented in Fig. 2, the angular dispersion of the XRD lines is ~5° (FWHM) in both ψ and ϕ directions, which indicates a moderate, but still acceptable, epitaxial film quality, with respect to the sputtering process. These values are, however, comparable to those reported by Chen *et al*.^18^ for thermal evaporation, which is a collisionless deposition process.

**Figure 3:**
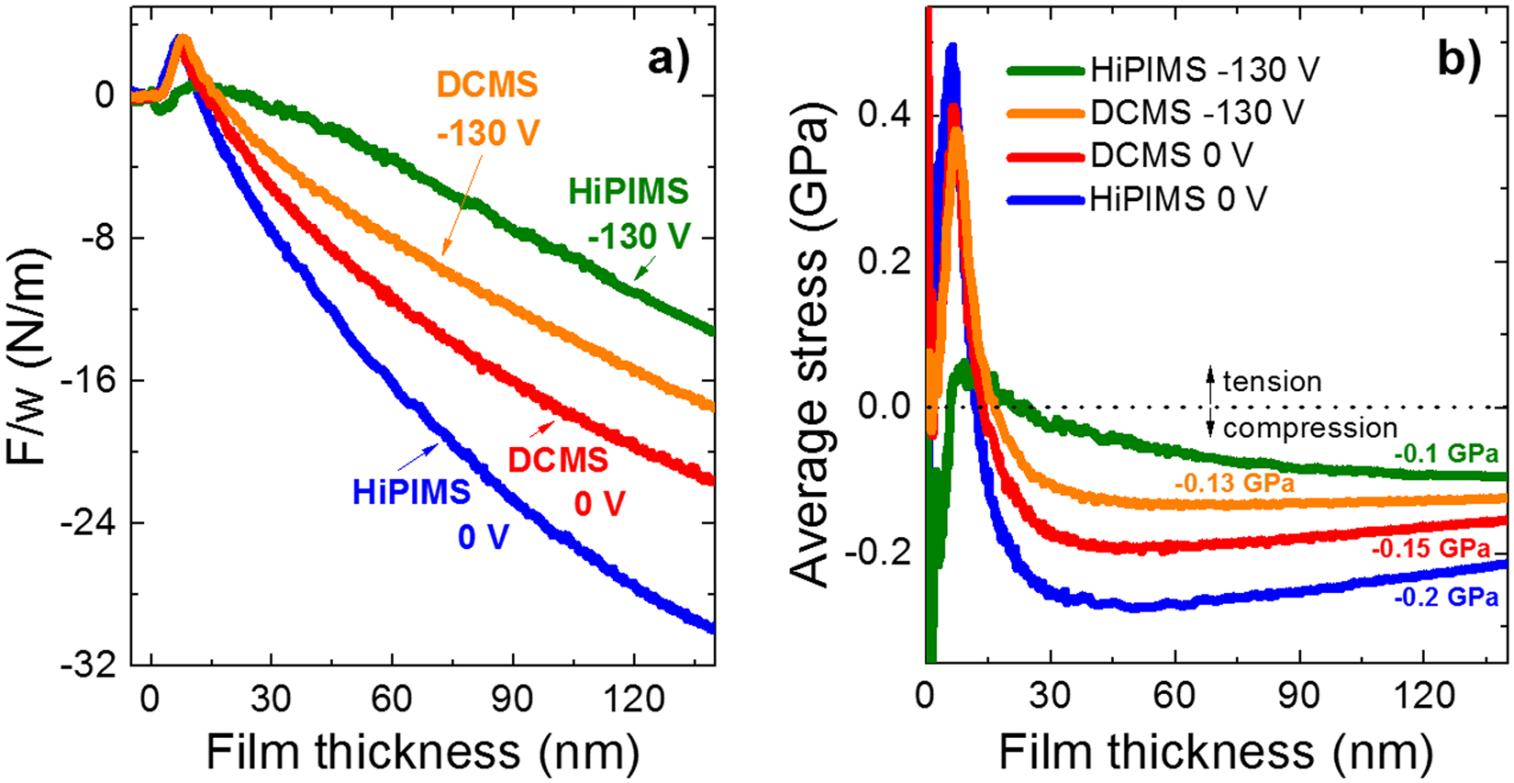
*In situ* intrinsic stress measurements during thin film growth. Evolution of (**a**) the film force per unit width, *F/w*, and (**b**) average stress, <σ>, as a function of film thickness for grounded (0 V) and −130 V bias DCMS and HiPIMS Cu films.

In addition, the intrinsic stress evolution during growth was monitored *in situ* using a multiple beam optical stress sensor (MOSS). The film force (Fig. 3(a)) and average stress (Fig. 3(b)) curves are compared for the DCMS and HiPIMS films at 0 V and −130 V bias. For the DCMS films (red and orange curves), a typical compressive-tensile-compressive (CTC) curvature change with the film thickness is obtained. The CTC behavior is commonly related to the Volmer-Weber growth mode, comprising island nucleation and growth (first compressive stage), island impingement and coalescence (tensile stage), and continuous film development (second compressive stage). The tensile peak maximum occurs for a film thickness of ~8 nm, which corresponds to the onset of film continuity once the coalescence stage is completed, as recently demonstrated by Abadias *et al*.^28^ for a series of metallic films grown on insulators in a Volmer-Weber mode. The average stress profile of the DCMS samples is displayed in Fig. 3(b): after a tensile maximum of 400 MPa, the stress rapidly decreases and becomes compressive, and reaches a constant value of −150 MPa (0 V) and −130 MPa (−130 V) with further film thickening (above 100 nm). The observation of a CTC behavior in the early stages of growth is consistent with literature data on stress evolution in Cu polycrystalline films^29–31^, though the critical thickness for film continuity and post-coalescence compressive stress magnitudes may vary depending on the kinetics and energetics of the deposition process^32^.

The HiPIMS Cu films deposited at 0 V bias exhibit a similar CTC behavior (Fig. 3(a), blue curve), but one can observe that larger compressive stress (−200 MPa) is developed in the post-coalescence stage (Fig. 3(b), beyond 15 nm film thickness). Although identical working pressure and average discharge power have been used, it is important to remember that the flux of film forming species arriving at the substrate differ between DCMS and HiPIMS. For the DCMS case, it consists of mainly Cu neutrals^33^ with typical energies in the range of a few eV or less, depending on process pressure and target-to-substrate distance. For HiPIMS, the vapor flux is characterized by a much higher fraction of ionized sputtered species. The ionized metal flux fraction, ${{\rm{\Gamma }}}_{{M}^{+}}/({{\rm{\Gamma }}}_{{M}^{+}}+{{\rm{\Gamma }}}_{M})$, where ${{\rm{\Gamma }}}_{{M}^{+}}$ is the metal ion flux to the substrate and Γ_*M*_ the metal neutral flux, typically reaches 80% or more during the peak of the discharge pulse for Cu sputtering at standard HiPIMS conditions^34^. When not applying a substrate bias, these Cu^+^ ions have an average kinetic energy of approximately 15–20 eV^35^, *i*.*e*., somewhat more energetic than the Cu neutrals. We believe that the slightly larger compressive stress for the HiPIMS film, as compared to the DCMS film, at 0 V bias, is due to this difference in the energetic bombardment of the growing film structure. It is known that energetic species will cause film densification and introduce point defects in the growing layer, which typically give rise to compressive stress^36, 37^. For Cu, molecular dynamics simulations have shown that 20 eV was sufficient energy to produce interstitial defects, up to several atomic planes below the surface^38^.

We now turn the discussion to the biased samples. An increasing applied bias voltage enables acceleration of Cu ions, but not neutrals. We do not see any significant change in the crystalline structure (Fig. 1), and we only see a small change on the stress levels (Fig. 3) of the DCMS films for 0 V and −130 V bias, due to the predominantly neutral flux. However, for the HiPIMS process at −130 V, a considerable energy increase of the bombarding Cu ions is expected. The energy provided by Cu^+^ ions increases from ~20 eV to ~130 eV, *i*.*e*., by a factor 6. This results not only in Cu implantation and sputter etching of the substrate surface (also discussed below concerning the TEM results) but also in favorable conditions for continuous epitaxial growth. For epitaxial growth the required migration and ordering of atoms on the film surface only occurs within a certain energy window, which is material dependent^39^. In the present case, the energy provided to the surface is shared between the Cu^+^ ions and the arriving Cu neutrals, increasing the surface mobility for both species. The additional energy provided suggests that surface mobility can indeed help to obtain the desired crystal arrangement. Furthermore, any thermal effects to the growth process were investigated in a separate experiment under similar process conditions using a passive thermal probe. It was found that the total energy influx to the substrate was always about 30% lower in HiPIMS compared to DCMS for the investigated Ar/Cu process at the same average discharge power (not shown here), and thus no additional substrate heating is expected. This result is mainly attributed to a lower total deposition rate in the HiPIMS case (see also Methods section), which reduces the energy contribution from the depositing species. However, although the total energy influx is lower, it is still found that 1.4 times more energy per deposited particle is achieved in HiPIMS as compared to DCMS, in line with previous investigations^40^.

**Figure 4:**
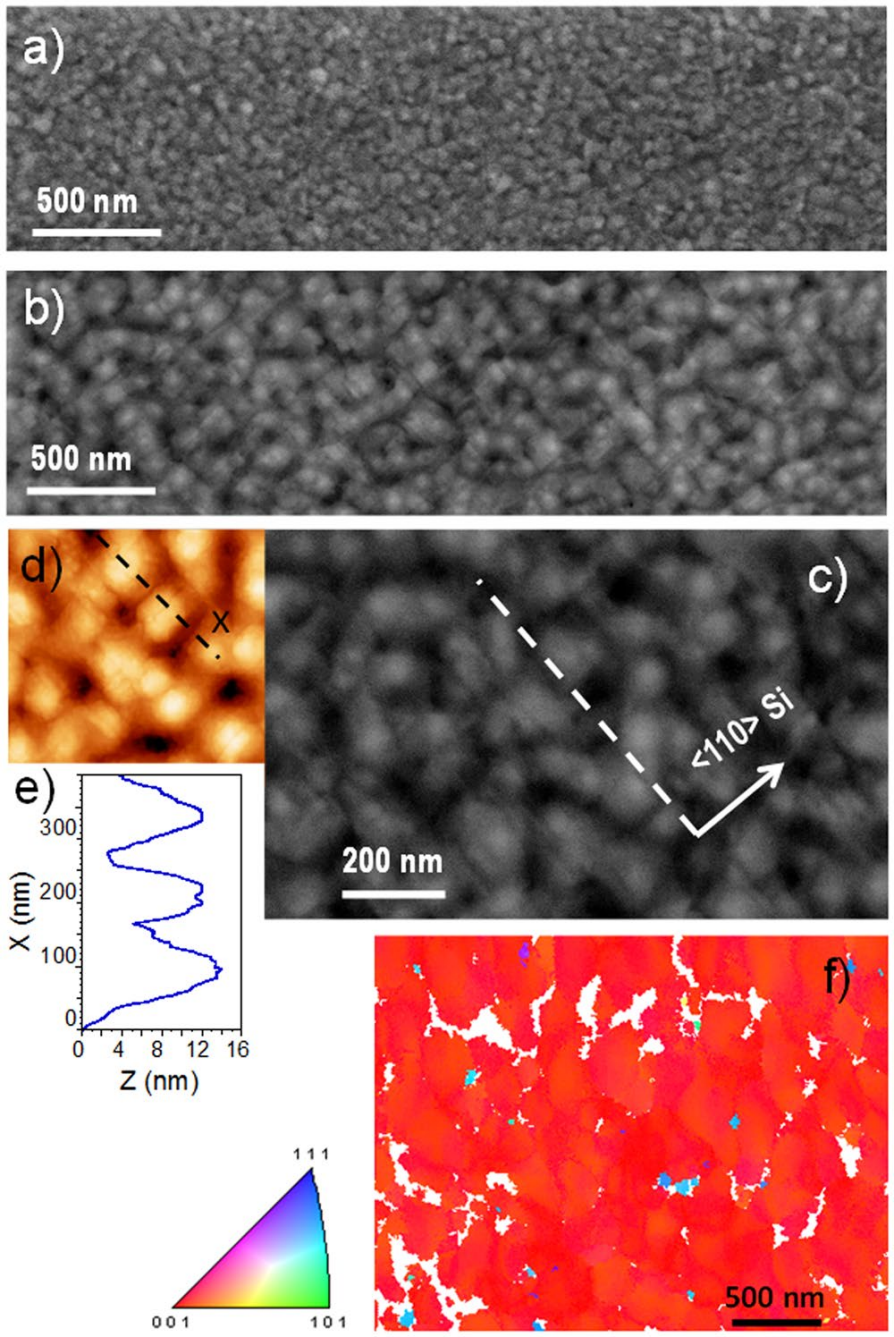
Microscopic characterization on the top-surface of Cu films. SEM micrographs (top view) of the surface morphology of (**a**) DCMS and (**b**) HiPIMS Cu films (~150 nm thick) grown at −130 V bias. (**c**) SEM and (**d**) AFM images at higher magnification showing the cubic domains network for the HiPIMS film. (**e**) Surface profile taken along the “x” direction (see dashed line in (**d**)). (**f**) Orientation map measured from EBSD for the Cu HiPIMS film at −130 V and corresponding color code. The slight variations in red hue correspond to max. 5–6° misorientation from the [001] axis.

In addition, it should be noted that such an energy increase of the bombarding species typically leads to larger compressive stress, as observed above when we compared HiPIMS and DCMS films at 0 V bias. However, the stress evolution of the Cu films deposited at −130 V shows the opposite behavior: the average stress saturates at only −100 MPa for the HiPIMS film (Fig. 3(b), green curve), which is lower compared to the −130 MPa obtained for the corresponding DCMS film (orange curve). For the biased HiPIMS sample, one can also notice that the early growth stage is distinctly affected: a sharp compressive transient is visible at the start of the deposition and a much broader tensile peak is established at a film thickness around 12 nm (Fig. 3(a)). Scanning electron microscopy (SEM) observations show that the Cu grain size is increased by a factor ~3 for the HiPIMS films (see Fig. 4(b)) deposited at −130 V compared to the DCMS case (see Fig. 4(a)) for films with the same average thickness (~150 nm). Noticeably, for biased HiPIMS film, the surface morphology consists of square-shaped mounds arranged in a rather periodic array, and aligned along two principal directions (see Fig. 4(c)) parallel to the <110> directions of the Si substrate, which corroborates the XRD observations for the epitaxial film. This morphology is also confirmed by atomic force microscopy (AFM) observations (Fig. 4(d)). The height profile, displayed in Fig. 4(e), shows a mound separation of ~130 nm, and height variations of 8–10 nm between valleys and tops. As a comparison, the average mound separation is ~50 nm for the DCMS Cu films deposited at −130 V bias. It may be concluded that the higher adatom mobility during the HiPIMS process at −130 V bias leads to a lower island density during the nucleation stage, and consequently to the development of larger grains. This explains why the onset of film continuity is delayed (12 nm instead of 8 nm) compared to the DCMS case. As most of the compressive stress arises from insertion of excess atoms into the grain boundary during deposition^37, 41^, it also explains why the HiPIMS Cu film, with larger grains and consequently a lower grain boundary density, exhibits a lower compressive stress compared to the DCMS Cu film. However, energetic ion bombardment also creates defects inside grains^37^, at the origin of larger microstrain and broadening of 200 XRD line (Fig. 1, green curve). Electron backscattering diffraction (EBSD) analysis confirms that the Cu grains of the biased HiPIMS sample share a common [001] out-of-plane orientation (see Fig. 4(f)), with an angular dispersion of max. 5–6°, except a few, smaller grains with other orientations. Pole figures, reconstructed from the EBSD maps (not shown here), match perfectly those obtained using XRD, confirming the epitaxial relationship between Cu and Si.

**Figure 5:**
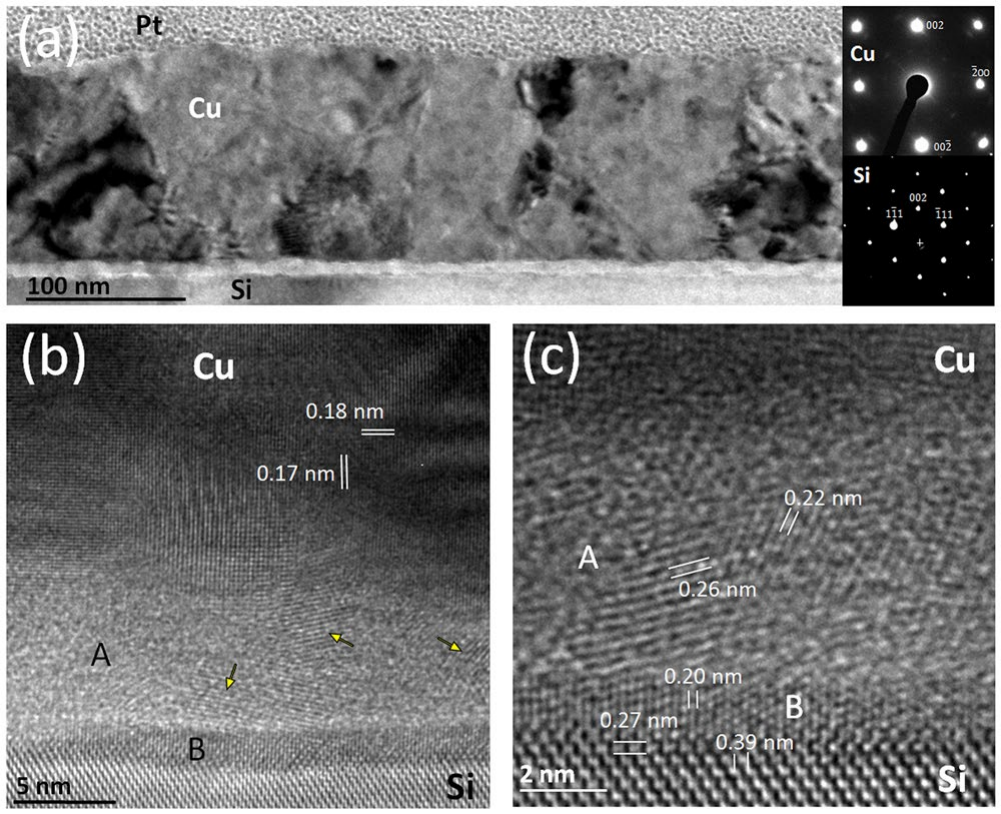
Cross-sectional TEM images of the HiPIMS Cu film grown at −130 V bias on Si(001) substrate. (**a**) Low magnification FTEM image; SAED patterns of the Cu layer and Si substrate are shown in the inset. (**b**) and (**c**) HR-TEM images of the Cu/Si interface region showing distinct interfacial layers, labelled A and B. Yellow arrows indicate crystalline domains in region A.

To further understand the epitaxial growth, transmission electron microscopy (TEM) measurements were carried out on the HiPIMS Cu film deposited at −130 V bias. A cross-sectional view of this sample is shown in Fig. 5(a). The main visible feature is a columnar grain growth of the Cu film with an average lateral grain size of ~100 nm. However, there is a large grain size distribution, since small grains co-exist and some tend to develop a V-shaped morphology with increasing film thickness. This behavior is clearly seen in a thicker film (400 nm) deposited at exactly the same conditions (see Supplementary Fig. S1)^42^. The presence of V-shaped columns suggests that grain size changes primarily at the surface^31^ and that bulk diffusion is rather limited. Another evidence of surface diffusion is noticed from the evolution of the intrinsic stress at the end of the deposition (see Supplementary Fig. S2) for the HiPIMS Cu films. The relaxation curves show a fast tensile rise, which saturates after a few minutes, with time constants of stress relaxation decreasing from ~40 s to ~20 s with increasing negative bias from 0 to −130 V. These values are typical of a surface-diffusion mediated relaxation process^43^, and indicates a faster mechanism under biased conditions.

The strong dark-bright straight fringes in Fig. 5(a) are associated with defects such as twins, dislocations and/or stacking faults, as typically observed for Cu films due to the low stacking fault energy of this metal^44^. The columns emerge at the surface with dome-like surface morphology, in good agreement with the AFM observations (Fig. 4(d)). One can observe the presence of bundling or grooving phenomena at the grain boundary, depending on misorientations between columns^45^. Locally, the grain-boundary grooves at the triple junction can reach 20 nm depth (see red arrow in Supplementary Fig. S1). The visible contrasts on the Si substrate are attributed to the TEM specimen preparation (ion beam cross-section thinning). At this magnification, the interface between the Cu film and the Si substrate appears straight. The bottom inset in Fig. 5(a) is a selected area electron diffraction (SAED) pattern taken from the Si substrate showing the <110> zone axis. The top inset in Fig. 5(a) corresponds to SAED pattern taken from a single Cu grain. This diffraction corresponds to a <100> zone axis of Cu, the growth direction being along [001], consistent with the epitaxial relationship derived from the XRD pole figure (Fig. 2(b)).

Figure 5(b) is a filtered high resolution (HR) TEM image close to the Cu/Si interface. The Cu layer is crystalline, with atomic plane contrasts visible in the in-plane and out-of-plane directions. Moiré fringes arise from the superposition of slightly disoriented grains along the thickness of the specimen. The measured atomic plane distances are in good agreement with the expected Cu {002} distance of 0.181 nm. However, a slight tetragonal deformation is observed and it is consistent with in-plane compressive stress (due to 6% misfit between Cu [110] and Si [100]). The interfacial layer consists of two regions with different contrasts, labelled A (~6 nm thick) and B (<2 nm thick). While the presence of well-defined atomic fringes is evidenced in layer B with dark contrast, layer A exhibits a brighter contrast and consists of a mixture of amorphous and nanocrystalline regions (crystalline domains are found locally, see yellow arrows).

A closer inspection of the interface (Fig. 5(c), HR-TEM image at higher magnification) shows that layer B displays lattice planes perpendicular to the interface. The measured distance between two atomic planes is 0.20 nm, which differs significantly from the interatomic spacing of Cu (0.181 nm). In layer A, however, several lattice planes contrasts are visible with different orientations and distances. Two distances are reported on Fig. 5(c), one, almost parallel to the interface, equals 0.26 nm and another, almost normal to the interface, equals 0.22 nm. It is important to note that these atomic planes contrasts are localized, and embedded in an amorphous or nanocrystalline contrast. This complex interface is similar to the interface observed by Echigoya *et al*.^46^ after annealing at 473 K a Cu film deposited by conventional magnetron sputtering on an atomically cleaned Si (001) substrate. At the interface, they observed a first layer, very close to our B layer contrast, composed by η”-Cu_3_Si, with a distance between two atomic planes contrast of 0.21 nm. At the top of this layer, they observed an amorphous phase, which ultimately leads to the formation of another silicide, ε-Cu_15_Si_4_, on the top of the amorphous layer. We cannot confirm that layer A consists of the same silicide, since local determination of the elemental composition at this scale is beyond the limit of our experimental set-up.

Last, we would like to emphasize that the chemical-physical mechanisms involved in the epitaxial growth of Cu on Si are still not completely understood, with several contradictory results reported in the literature, as discussed in more detail elsewhere^17^. In the typical case of Si substrates atomically cleaned by surface heating, HF etching, or Ar^+^ bombardment, a pure Si surface is available when the Cu deposition starts. However, it has been generally recognized that the Cu/Si interface is complicated by the intermixing of Cu and Si during deposition. Cu has a strong tendency to react with Si, often forming the copper silicide η”-Cu_3_Si, as described above concerning layer B, which can be easily oxidized at room temperature according to the Cu_3_Si + O_2_ → SiO_2_ + 3Cu reaction route^10, 47–49^. Generally, the Cu/Si interface is presented as a mixed structure composed of different copper silicides or amorphous phases, and their chemical nature is not well defined^10, 11, 13, 16, 46^, which we believe is key when trying to understand the formation of the interlayers. In our HiPIMS case at −130 V bias, such interlayer is likely even more complex, since Cu implantation, SiO_2_ etching, and Cu deposition take place at the same time during the early stage of film growth, due to the energetic flux of Cu^+^ ions. It may be that a fraction of the energetic Cu^+^ ions are implanted below the native SiO_2_ oxide and thereby form a copper silicide (Fig. 5(c), layer B). SRIM calculations^50^ of the ion damage into a SiO_2_(2 nm thick)/Si(substrate) system support this scenario: the mean projected range, *R*_p_, of Cu^+^ ions with 130 eV is 1.7 ± 0.3 nm, showing that most Cu^+^ ions will penetrate into the native SiO_2_ oxide layer, and that a non-negligible fraction will reach the SiO_2_/Si interface and react with Si atoms from the substrate. In parallel, the ionic flux will also interact with the SiO_2_ causing intermixing in combination with sputter etching, and thereby forming a complex mixed layer (Fig. 5(c), layer A) containing nanocrystalline and amorphous compounds of Si, O and Cu. SRIM results indicate a significant preferential sputtering of O atoms (the sputtering yield of O and Si atoms are 0.148 and 0.017 atom/ion, respectively). Re-sputtering of the Cu layer is also quite substantial during HiPIMS deposition under −130 V bias, as confirmed by a decrease of the deposition rate by a factor 1.7 (see Methods section), in good agreement with SRIM calculations. Further experiments would be required to unravel the underlying mechanisms of subsequent Cu crystal growth over these complex transition interlayers.

## Conclusions

In this work, it has been shown that Cu (001) films can be epitaxially grown on Si (001) wafers covered with a native oxide layer, by using a single-step HiPIMS deposition process and a substrate bias of −130 V, at room temperature. Until now, this has not been possible to achieve by any deposition method without prior substrate cleaning, such as chemical or plasma etching. The Cu/Si heteroepitaxial growth followed the Cu(001) [100]//Si(001) [110] orientation through a complex interface composed of different copper silicides or amorphous phases. The Cu surface morphology exhibited a regular pattern consisting of square-shaped mounds with a lateral size of typically 150 nm. The Cu grain size increased by a factor ~3 for the HiPIMS film deposited at −130 V compared to films deposited by conventional DCMS at otherwise similar deposition conditions. It is likely due to the higher adatom mobility during the HiPIMS process, which leads to a lower island density during the nucleation stage, and consequently to the development of larger grains. Such grain growth also results in a lower compressive stress due to a lower grain boundary density. It is therefore concluded that the strategy used in the present study could potentially open up more efficient routes for depositing epitaxial films for nano-ranged downscaled electrical, electronic, and magnetic devices.

## Methods

### Thin film growth

Cu films ~150 nm thick were deposited by two different magnetron sputtering processes at room temperature on Si (001) wafers, 100 µm thick, covered with a native oxide (SiO_x_), without any prior substrate cleaning process. We have also deposited films ~30 nm thick at the same experimental conditions, only to obtain the deposition rates, and one thicker film (400 nm) to observe microstructure development. The depositions were carried out in a high vacuum chamber (base pressure <10^−5^ Pa) equipped with a bottom-mounted, 7.5 cm-diameter high purity Cu target operated by either a DC power supply (SR1.5-N-1500, Technix, France) or a HiPIMS power supply (HiPSTER 1, Ionautics, Sweden). The target was located at 18 cm from the top-mounted substrate holder, which was polarized by a negative DC bias power supply. More details on the experimental setup can be found elsewhere^51^. The substrate bias voltage was varied from 0 to −160 V for the deposited Cu films grown in both DC magnetron sputtering (DCMS) and HiPIMS mode. In the present work, we focus mainly on the coatings deposited at bias voltages of 0 and −130 V, since these coatings exhibited the greatest differences. The total pressure was kept constant at 0.5 Pa using Ar as an inert process gas, and the average target power was fixed at 200 W for all studied samples. For the DCMS depositions, the target voltage during the discharge was −470 V and the target current was 430 mA. For the HiPIMS depositions, square voltage pulses of −800 V with 40 µs pulse-on time and a pulse frequency of 250 Hz (duty cycle of 1%) were applied to the cathode. The average peak current value was 43 A (peak target current density of ~1 A cm^−2^).

Different deposition times were used to obtain the same final film thickness, depending on the deposition rate obtained in each mode of operation. The deposition rate for the HiPIMS samples decreased from 0.19 nm s^−1^ (for a grounded substrate) to 0.11 nm s^−1^ when applying a bias voltage of −130 V. The high negative bias voltage leads to an increased kinetic energy of the Cu ions bombarding the film surface. It promotes a more efficient sputter etching of the growing film, since the sputter yield increases with increased kinetic ion energy^52^, resulting in a lower deposition rate. For the DCMS case, the deposition rate is not dependent on bias voltage, being equal to 0.36 nm s^−1^. In this case, Cu atoms are the dominant species forming the film, and thereby, not influenced by accelerating voltages applied to the substrate.

### Thin film characterization

The real-time stress evolution was determined *in situ* from the measurement of the substrate curvature change during film deposition, using a multiple beam optical stress sensor (MOSS) designed by k-Space Associates, Inc, USA, with a curvature resolution of 2 × 10^−4^ m^−1^. The force in the film per unit width, *F*/*w*, given by the product between the average stress σ and film thickness *h*, was calculated from the measured curvature Δκ using the modified Stoney equation $\frac{F}{w}=\sigma h=\frac{1}{6}{Y}_{s}{h}_{s}^{2}{\rm{\Delta }}\kappa$, where *h*_*s*_ is the substrate thickness and *Y*_*s*_ is the biaxial modulus of the substrate, which was assumed to be equal to 180.5 GPa for (001) single crystal Si wafers^53^. The film thickness was determined *ex situ* by X-ray reflectometry (XRR) for films ~30 nm thick using a Seifert XRD3000 diffractometer in parallel beam configuration, and from scanning electron microscopy (SEM) imaging of cross-sectional samples prepared by focused ion beam (FIB) for films ~150 nm thick, using a FEI-Helios NanoLab G3 Dual-Beam microscope operating at 5 keV.

The crystallographic orientation was determined by X-ray diffraction (XRD) including conventional θ–2θ scans carried out on a D8 Bruker AXS diffractometer operating in the Bragg-Brentano configuration at λ = 0.15418 nm wavelength and pole figure measurements using a four-circle XRD3000 Seifert diffractometer operating in point focus geometry. Complementary electron backscatter diffraction (EBSD) analysis was performed using the FEI-Helios Dual Beam platform, operating at 10 keV and 11 nA and equipped with a EDAX-Hikari camera with an acquisition rate of 100 images per second and a 10 nm step size. The collected EBSD signal was treated using the OIM software, assuming misorientation angles lower than 2° within a grain.

The surface morphology of the films was analyzed immediately after deposition by atomic force microscopy (AFM) using a multimode Digital Instrument microscope operating in tapping mode at ambient air. Microstructural characteristics of the films in plane-view were investigated using a JEOL 7001F-TTLS SEM microscope operating at 10 kV, while cross-sectional lamellae, prepared by FIB, were used for transmission electron microscopy (TEM). For the ~150 nm thick HIPIMS Cu film deposited at −130 V bias, the cross-section was extracted along the <110> zone axes of the Si substrate. After the deposition of a protective Pt layer, the initial milling was done using 30 keV Ga ions and a current of 10 nA. To achieve electron transparency, the ion beam energy was reduced to 1 keV and the current to 10 pA. TEM observations were performed using a JEOL 2200FS microscope equipped with a field emission gun and operated at 200 kV. Images were acquired with elastic electron beams using a 7 eV width filter placed around the zero loss peak.

## Electronic supplementary material

SupplementaryInformation
