# Supplementary material for: Epitaxial growth of Cu(001) thin films onto Si(001) using a single-step HiPIMS process
Source: Sci Rep. 2017 May 10;7:1655. doi: 10.1038/s41598-017-01755-8 (PMC5431785; doi:10.1038/s41598-017-01755-8)
Supplement: Supplementary file 1 — SupplementaryInformation [file 41598_2017_1755_MOESM1_ESM.pdf]

Supplementary Information

Original article – *Scientific Reports*

Epitaxial growth of Cu(001) thin films onto Si(001) using a single-step HiPIMS process

Felipe Cemin[1,*], Daniel Lundin[1], Clarisse Furgeaud[2], Anny Michel[2], Guillaume Amiard[2], Tiberiu Minea[1] & Gregory Abadias[2]

[1] Laboratoire de Physique des Gaz et des Plasmas (LPGP), UMR 8578 CNRS, Université Paris-Sud, 91405 Orsay, France

[2] Institut Pprime, Département Physique et Mécanique des Matériaux, UPR 3346 CNRS, Université de Poitiers, 86962 Chasseneuil-Futuroscope, France

*E-mail: felipe.cemin@u-psud.fr

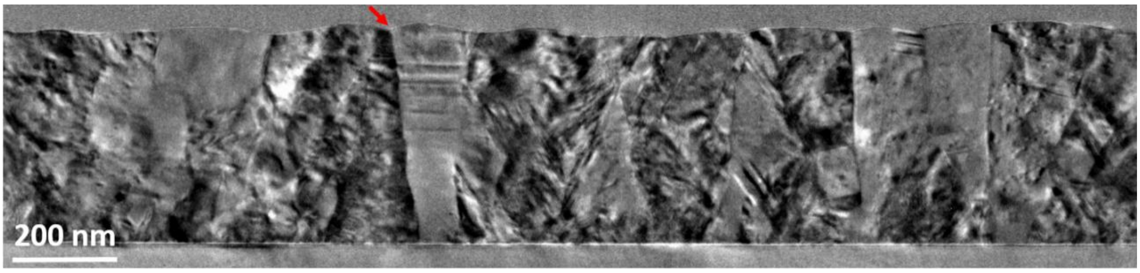

Supplementary Figure S1. **Cross-sectional TEM image of the Cu film (400 nm thick) deposited by HiPIMS.** The substrate (Si(001)) was biased at −130 V during Cu deposition. Some grains tend to develop a V-shaped morphology with increasing film thickness. The red arrow indicates grooving phenomena at the grain boundary.

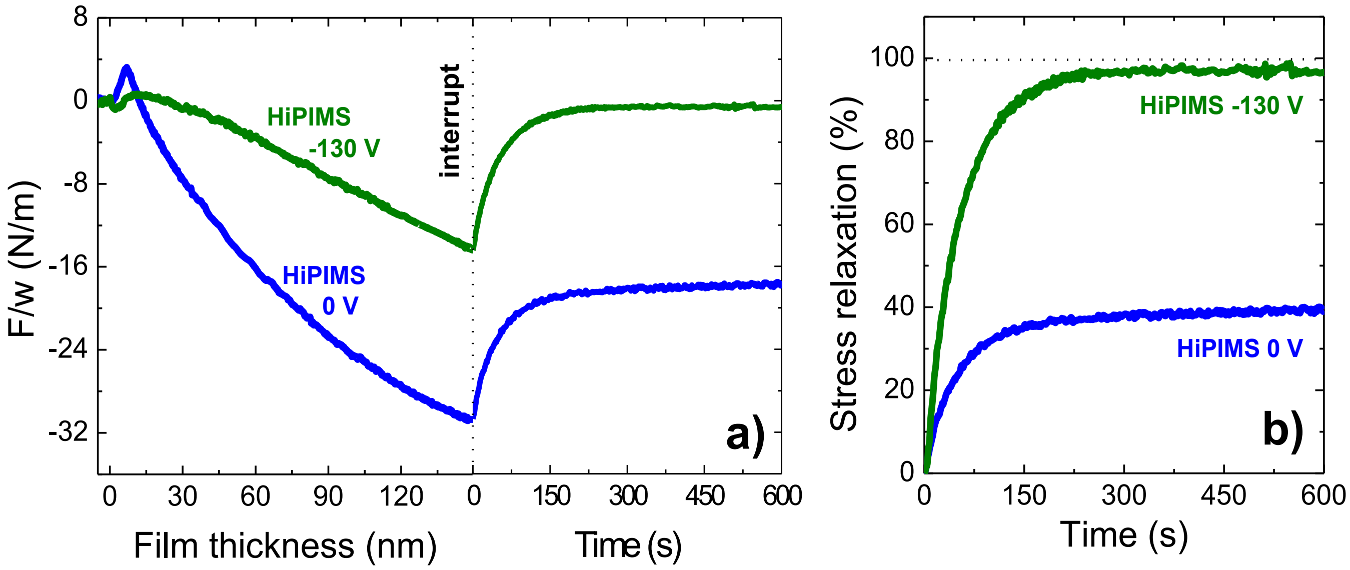

Supplementary Figure S2. ***In situ* intrinsic stress measurements during thin film growth.** (**a**) Evolution of the film force per unit width, *F/w*, during and after deposition of Cu films (150 nm thick) by HiPIMS using grounded (0 V) and biased (−130 V) Si(001) substrates. (**b**) Relative stress relaxation, normalized to the magnitude of the compressive stress developed from the tensile peak till the growth interruption.
